# Whole-Exome Sequencing Followed by dPCR-Based Personalized Genetic Approach in Solid Organ Transplantation: A Study Protocol and Preliminary Results

**DOI:** 10.3390/mps8020027

**Published:** 2025-03-04

**Authors:** Mirgul Bayanova, Aidos Bolatov, Dias Malik, Aida Zhenissova, Aizhan Abdikadirova, Malika Sapargaliyeva, Lyazzat Nazarova, Gulzhan Myrzakhmetova, Svetlana Novikova, Aida Turganbekova, Yuriy Pya

**Affiliations:** 1 Genetic Unit, Department of Laboratory Medicine, Pathology and Genetics, “University Medical Center” Corporate Fund, Astana 010000, Kazakhstan; mirgul.bayanova@umc.org.kz (M.B.); dias.malik@umc.org.kz (D.M.); zhenissova1994@gmail.com (A.Z.); a.abdikadirova@umc.org.kz (A.A.); malika.s@umc.org.kz (M.S.); layzzat.nazarova@umc.org.kz (L.N.); 2 School of Medicine, Shenzhen University, Shenzhen 518060, China; 3 School of Medicine, Astana Medical University, Astana 010000, Kazakhstan; 4 Clinical Academic Department of Cardiology, “University Medical Center” Corporate Fund, Astana 010000, Kazakhstan; mirzakhmetovaguljan@gmail.com; 5 Clinical Academic Department of Cardiac Surgery, “University Medical Center” Corporate Fund, Astana 010000, Kazakhstan; s.novikova@umc.org.kz (S.N.); yuriy.pya@umc.org.kz (Y.P.); 6 HLA-Laboratory, Scientific-Production Center of Transfusiology, Astana 010000, Kazakhstan; aidaturganbekova84@gmail.com

**Keywords:** transplantation, genetic testing, whole-exome sequencing, pharmacogenetics, dd-cfDNA, clinical utility

## Abstract

Genetic profiling and molecular biology methods have made it possible to study the etiology of the end-stage organ disease that led to transplantation, the genetic factors of compatibility and tolerance of the transplant, and the pharmacogenetics of immunosuppressive drugs and allowed for the development of monitoring methods for the early assessment of allograft rejection. This study aims to report the design and baseline characteristics of an integrated personalized genetic approach in solid organ transplantation, including whole-exome sequencing (WES) and the monitoring of dd-cfDNA by dPCR. Preliminary results reported female recipients with male donors undergoing two pediatric and five adult kidney and three heart transplantations. WES revealed a pathogenic mutation in *RBM20* and VUS in *TTN* and *PKP2* in heart recipients, while kidney donors presented mutations in *UMOD* and *APOL1* associated with autosomal-dominant kidney diseases, highlighting the risks requiring the long-term monitoring of recipients, donors, and their family members. %dd-cfDNA levels were generally stable but elevated in cadaveric kidney recipient and one pediatric patient with infectious complications and genetic variants in the *ABCB1* and *ABCC2* genes. These findings highlight the potential of combining genetic and molecular biomarker-based approaches to improve donor–recipient matching, predict complications, and personalize post-transplant care, paving the way for precision medicine in transplantation.

## 1. Introduction

Genetic factors play an important role in transplant tolerance and rejection after the transplantation of solid organs [1]. Technological advances in molecular research have made significant progress toward personalized transplantation medicine [2]. Various approaches from HLA typing to comprehensive omics studies have been proposed to study the etiology of the last stage of organ failure before transplantation, the role of various polymorphisms in transplant tolerance, the pharmacogenetics of immunosuppressive drugs and the search for noninvasive biomarkers to assess the risk and differentiate the type of transplant rejection.

Previously, using the GWAS method identified several SNPs that were associated with new-onset diabetes after transplantation, cutaneous squamous cell carcinoma, basal cell carcinoma and nonmelanoma skin cancer developed after transplantation, acute renal rejection, and cardiovascular diseases in kidney transplantation (KTx) [1]. At the same time, the NGS has made it possible to develop cost-effective approaches in genomic profiling. Thus, Tantisattamo et al. found autosomal dominant Alport syndrome in the post-donation period and recommended the incorporation of pre-donation genetic testing into living kidney donor evaluation [3]. Moreover, it is strongly recommended to consider the possibility of whole-exome or Sanger sequencing-based genetic testing of potential donors with a family history of suspected monogenic forms of end-stage kidney disease, as described in the KDIGO guidelines [4,5]. The diagnostic usefulness of genetic tests allows us to take a fresh look at the clinical situation in most families with pre-planned KTx [6]. Redondo et al. reviewed several immune system-associated genes’ polymorphisms as a risk for viral infection after solid organ transplantation and concluded that genetic susceptibility testing may improve personalized medicine and contribute to minimizing the risk of viral infection after transplantation [7]. Cardiomyopathy genes panel-targeted NGS among heart transplant patients allowed Boen et al. to find a positive genotype in 39.6% of family members, of which 52.6% had heart rhythm abnormalities; although at the time of examination, all of them were asymptomatic [8]. The detection of pathogenic variants among heart transplant recipients’ family members might prevent sudden cardiac death or alter heart transplantation or VAD implantation clinical decisions [9]. Thus, genomic profiling may have a potential clinical role for both the recipient and donor.

In 2016, the USA Food and Drug Administration (FDA) meeting on patient-focused drug development and medication adherence highlighted the need for simplified and individualized immunosuppressive therapy in solid organ transplant recipients [10,11]. Pharmacogenetics, a key aspect of personalized and precision medicine, integrates an individual’s genetic profile with other clinical characteristics to optimize treatment strategies. This approach can enhance the prediction of drug response and reduce the risk of adverse reactions, while also being a cost-effective solution [11,12,13]. Several organizations, including the Clinical Pharmacogenetics Implementation Consortium, the Royal Dutch Association for the Advancement of Pharmacy, the Canadian Pharmacogenomics Network for Drug Safety, and the French National Network of Pharmacogenetics, have developed pharmacogenetic-based drug dosing guidelines. These guidelines provide valuable insights into interpreting *CYP3A5*/*CYP3A4* genotype results for tacrolimus dosing adjustments [14,15]. Additionally, genes such as *TPMT*, *UGT1A9*, *UGT2B7*, *IMPDH2*, *MRP2*, *ABCB1*, *POR*, and *CYP3AP1* have been implicated in the pharmacogenetics of various immunosuppressive drugs, including azathioprine, cyclosporin, mycophenolic acid, sirolimus, and everolimus [16,17,18,19,20,21,22,23]. In Kazakhstan, according to the study among KTx recipients, 61.25% (49 of 80) of patients were homozygotes to CYP3A5*3*3 (non-expressers), which proves that genotype-based dosing may be the key factor in the determination of preferred doses of tacrolimus [24].

Despite advancements in immunosuppressive therapy, allograft rejection remains the leading cause of solid organ transplant dysfunction [25]. Various functional parameters are utilized to monitor graft function post-transplantation, with biopsies considered the gold standard for assessing allograft health. However, routine biopsy monitoring is not ideal due to its invasive nature, high cost, and the associated risks of complications [26,27]. Over the past two decades, various studies have focused on finding an accurate noninvasive biomarker of allograft rejection. One of these biomarkers, donor-derived cell-free DNA (dd-cfDNA), may provide windows of opportunity to intervene early and before irreversible allograft injury [28,29]. However, randomized controlled trials and cost-effectiveness studies are necessary to validate the benefits and to guide the ideal incorporation of dd-cfDNA into routine clinical practice [28,30].

Genetic studies provide valuable insights into both the treatment and post-transplant monitoring of patients, as well as the underlying etiology of diseases affecting recipients and their families. This study aims to present a detailed protocol for integrating whole-exome sequencing (WES), pharmacogenetic analysis, and digital PCR-based donor-derived cell-free DNA (dd-cfDNA) quantification as a noninvasive, personalized approach to solid organ transplantation medicine. Given the lack of standardized genetic diagnostics in solid organ transplantation in Kazakhstan beyond HLA typing, this study seeks to establish a scientific and clinical framework for incorporating molecular genetic techniques into routine post-transplant management. To demonstrate the feasibility and broad applicability of this approach, we present preliminary findings from both kidney and heart transplantation. The inclusion of both transplant types allows us to assess the suitability of genetic and molecular biomarker-based strategies across different solid organ transplantation contexts, providing valuable initial data on genetic risk factors, pharmacogenetics, and noninvasive rejection monitoring. These preliminary results lay the groundwork for future large-scale studies and the clinical integration of personalized transplant medicine.

## 2. Materials and Methods

### 2.1. Study Design

The current prospective, observational, longitudinal study will recruit at least 40 adult and 15 pediatric kidney and 30 heart transplantation pairs of donors and recipients at the University Medical Center CF and National Research Oncology Center (Astana, Kazakhstan). Genetic diagnostic methods will consist of two stages. In the first stage, whole-exome sequencing (WES) of genomic DNA (gDNA) isolated from the donor and recipient will be provided. After bioinformatic analysis, the recipient’s genomic data will be used for pharmacogenetic studies and to study the etiology of diseases, followed by the genetic counseling of recipient family members. At the same time, the donor’s genomic data are planned to be used to assess the genetic risk of hereditary diseases of the transplanted organ, followed by genetic counseling of recipient family members, if necessary. The comparison of the genomic data of the donor and recipient makes it possible to identify unique and shared SNPs between them for the subsequent assessment of the ratio of dd-cfDNA in the next stage. In the second stage, it is planned to determine the ratio of dd-cfDNA using allele-specific digital PCR (dPCR).

**Figure 1:**
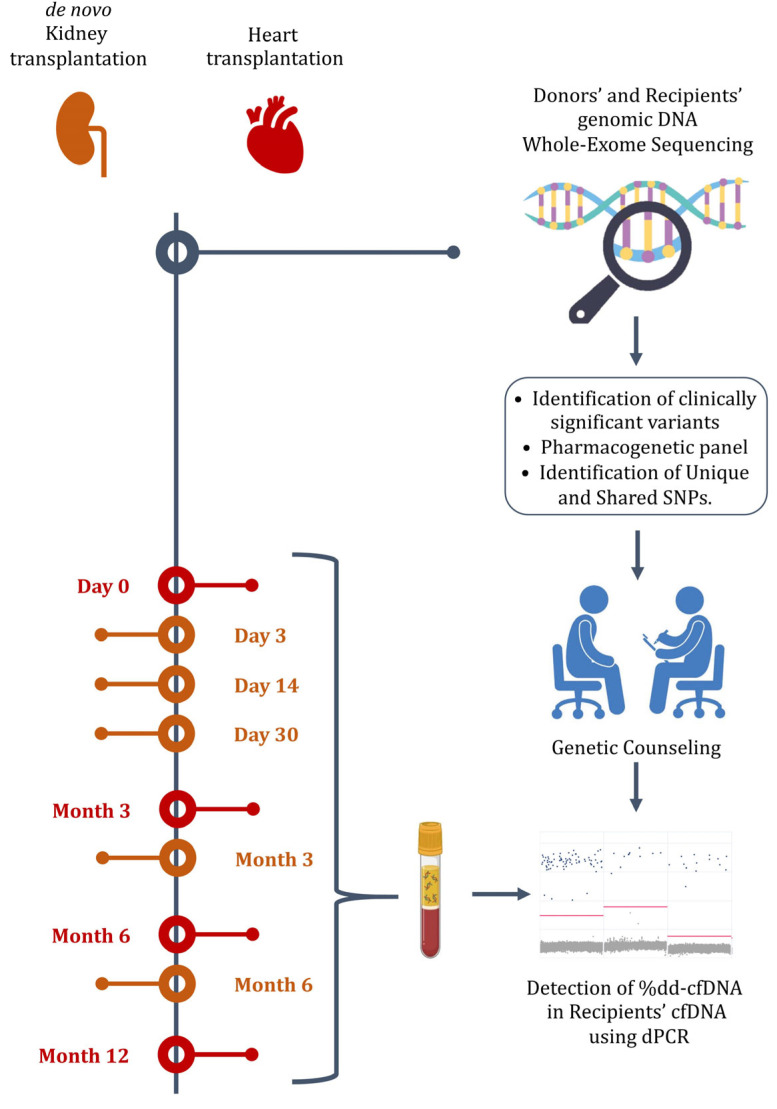
Study design.

The determination of dd-cfDNA will be performed 4 times for patients who have undergone heart transplantation (HTx) in the last 10 years (due to the relatively small number of heart transplantations) and 5 times for primary patients after KTx during the study period. So, for patients after HTx, dd-cfDNA level determination will be carried out according to the periods illustrated in Figure 1 or when clinical symptoms arise, and for patients after KTx, according to the following scheme: on day 3, on day 14, and on day 30 and 3 and 6 months after transplantation. Study participants with signs of allograft rejection will have additional dd-cfDNA determination.

### 2.2. Study Population and Eligibility Criteria

Donors and recipients of de novo adult and pediatric KTx, as well as recipients after HTx over the past 10 years, will be included in the study with informed written consent from the patient or one of their legal representatives. The gDNA of the heart donors will be obtained from the Research and Production Center of Transfusiology. Participants’ recruitment and material collection began in October 2023 and is scheduled to be completed in April 2025.

### 2.3. Participant Safety

All risks to the participants in the study were mitigated by ensuring that all recruitment and data collection were managed by appropriately trained and experienced research staff. Recruitment staff were research physicians and nurses with extensive experience in research and clinical practice. The data were stored in a secure setting, and data linkage software was adequately protected to maintain security and privacy.

### 2.4. Genomic and Cell-Free DNA Extraction

gDNA isolation from both the donor and recipient will be conducted at the start of the study. gDNA will be extracted from peripheral blood samples (100 µL–1 mL) using the PureLink™ Genomic DNA Mini Kit (Thermo Fisher Scientific Inc., Waltham, MA, USA), following the manufacturer’s protocol.

For cell-free DNA extraction, 10 mL of venous blood samples will be collected in cell-free DNA BCT tubes (©STRECK, La Vista, NJ, USA). Venous blood samples will be centrifuged within 2 h of collection at 1600× *g* for 20 min at room temperature. The plasma will be re-centrifuged at 16,000× *g* for 10 min at room temperature. The full plasma supernatant will be stored at −80 °C until cell-free DNA extraction. cfDNA will be extracted with the QIAamp Circulating Nucleic Acid Kit (Qiagen, Hilden, Germany) using the QIAvac Vacuum System (Qiagen, Hilden, Germany), following the manufacturer’s protocol. The final elution volume of cfDNA will be 100 µL.

Concentrations and ratios of A260/A280 in gDNA and cfDNA extracts will be determined using a NanoDrop ™ spectrophotometer One (Thermo Fisher Scientific Inc., Waltham, MA, USA). The isolated gDNA and cfDNA will be stored at a temperature of −80 °C.

The concentrations and ratios of A260/A280 in gDNA will be determined using a NanoDrop ™ spectrophotometer One (Thermo Fisher Scientific Inc., Waltham, MA, USA). The concentration of cell-free DNA was measured using the Qubit^®^ 3.0 Fluorometer (Thermo Fisher Scientific Inc., Waltham, MA, USA). The isolated gDNA and cfDNA will be stored at a temperature of −80 °C. Sample integrity will be monitored periodically, and storage conditions will adhere to standard biobanking protocols to maintain DNA quality for downstream analyses.

### 2.5. Whole-Exome Sequencing

Library preparation and NGS-based WES were carried out using the Twist Human Core Exom (+RefSeq) Kit (with >70x coverage on target, 6Gb/sample) on NovaSeq 6000 (Illumina, San Diego, CA, USA), according to the manufacturer’s instructions in Macrogen Inc. (Seoul, Republic of Korea). The quantity of gDNA was measured using the QuantiFluor^®^ dsDNA System (Promega, Madison, WI, USA) on a Victor Nivo Multimode Microplate Reader (PerkinElmer, Waltham, MA, USA). To assess the integrity of gDNA, the Agilent Technologies 2100 Bioanalyzer (Agilent, Santa Clara, CA, USA) or Agilent 4200 TapeStation (Agilent, Santa Clara, CA, USA) was utilized. The DNA Integrity Number (DIN) was used as an indicator of DNA fragmentation, providing a numerical assessment of DNA quality based on the size distribution of DNA fragments. DNA libraries were validated using the Agilent Technologies 2100 Bioanalyzer with the DNA 1000 chip and Illumina qPCR, according to the standard kits protocol. Sequencing data generated processed base calling using real-time analysis (RTA) software. The resulting binary base call (BCL) files were converted into FASTQ format using the Illumina bcl2fastq v2.20.0 software. Paired-end reads were aligned to the human reference genome (hg38, UCSC) using the Burrows–Wheeler aligner (BWA-MEM) algorithm. The aligned sequencing reads were further processed using base quality score recalibration (BQSR) to correct potential sequencing errors and improve variant calling accuracy. Single nucleotide variants (SNVs) and insertions/deletions (InDels) were identified using the HaplotypeCaller tool from the Genome Analysis Toolkit (GATK).

Identified causal genetic variants were described following the nomenclature guidelines of the Human Genome Variation Society (http://www.hgvs.org/mutnomen (accessed on 25 October 2024)) and the 5-level classification system recommended by the American College of Medical Genetics and Genomics and the Association for Molecular Pathology (ACMG/AMP).

Genetic variants associated with the etiology of end-stage organ disease will be validated using allele-specific digital PCR in trios (if possible). The analysis will be conducted on QIAcuity One (Qiagen) using the QIAcuity PCR Master Mix (Qiagen, Germany) and EvaGreen PCR Master Mix (Qiagen, Germany), following the manufacturer’s instructions and guidelines. Custom-designed allele-specific primers will be used for targeted variant detection.

### 2.6. Pharmacogenetic Analysis

According to the national protocol in Kazakhstan for the management of transplant patients, the immunosuppressive drug tacrolimus is routinely prescribed. In accordance with the guidelines set forth by the Clinical Pharmacogenetics Implementation Consortium (CPIC), the Dutch Pharmacogenetics Working Group (DPWG), and the Royal Dutch Pharmacists’ Association (RNPGx), based on literature review and data from PharmGKB for tacrolimus therapeutic drug monitoring (TDM), single nucleotide polymorphisms (SNPs) in the *CYP3A5* (rs55817950, rs776746, rs10264272, rs28383479, rs41303343), *CYP3A4* (rs35599367, rs28371759, rs67666821, rs4646438, rs138105638, rs4646437, rs2242480, rs4986910, rs2740574), *ABCB1* (rs1045642, rs9282564, rs2229109, rs2032582, rs1128503), *ABCC2* (rs717620, rs3740066), *C6* (rs9200, rs10052999), *CAPN10* (rs5030952), *CRTC2* (rs8450), *CTLA4* (rs4553808), *CYP2J2* (rs890293), *FOXP3* (rs3761548), *HSD11B1* (rs846908, rs4844880, rs846910), *IL19* (rs1800896, rs1800871, rs1800872), *IL18* (rs5744247, rs1946518), *IL3* (rs181781), *KCNJ11* (rs5219), *KCNQ1* (rs2237895), *NOD2* (rs2066844), *NR1I2* (rs3814055, rs2276707), *POR* (rs1057868), *PPARA* (rs4253728, rs4823613), *SUMO4* (rs237025), *TCF7L2* (rs290487, rs7903146), and *TLR4* (rs1927907) variants were analyzed [14,31,32,33,34].

### 2.7. Determination of dd-cfDNA%

Donors’ and recipients’ WES data were examined to identify unique SNPs-homozygous variants. The primary criteria for selecting SNPs include high-confidence scores, variant quality, quality-by-depth metrics, and sufficient sequencing depth, as high-quality scores help minimize false positives in sequencing results. Allele-specific primers were designed to position the SNP at the 2nd or 3rd nucleotide from the 3′-end of the primer. Primer design was carried out using Primer3Plus, with specificity assessed through in silico PCR simulations in the UCSC Genome Browser. Primer characteristics adhered to the following parameters: primer length of 18–27 bp, melting temperature (Tm) between 58–62 °C, amplicon length of 80–130 bp, GC content of 30–70%, and uniform Tm values within 2 °C across primers. Secondary structures, such as self-dimers, hetero-dimers, and hairpins, were avoided, as well as mismatches to the template sequence, following the manufacturer’s recommendations.

The QIAcuity One (Qiagen, Germany) was used for dd-cfDNA level determination by allele-specific dPCR using the QIAcuity EG PCR Kit (Qiagen, Germany). The concentration of donor-specific alleles (Cd) and the concentration of recipient-specific alleles (Cr) are planned to be calculated using the QIAcuity Software Suite (Qiagen, Germany), and the fraction of donor-specific alleles (dd-cfDNA%) is planned to be estimated using the following calculations (1):

(1) $$\mathrm{dd-cfDNA}\%=\frac{C_{d}}{C_{r}+C_{d}}*100\%$$

To validate dd-cfDNA% data from allele-specific dPCR, quantified Y-chromosome-specific DNA fragments analysis using the Investigator Quantiplex Pro Kit (Qiagen, Germany) will be utilized for male donor and female recipient pairs. The PCR reaction mixture will be consisted of 10 µL QIAcuity PCR Master Mix (Qiagen, Germany), 10 µL Primer Mix FQ from the Quantiplex Pro Kit, 10 µL nuclease-free water, and 10 µL cfDNA, resulting in a total reaction volume of 40 µL. Each sample will then load into a QIAcuity Nanoplate 26k 24-well plate (Qiagen, Germany) for digital PCR analysis. The preliminary results demonstrated data on Y-chromosome-specific DNA fragment analysis. The calculation to measure dd-cfDNA% based on Y-chromosome-specific DNA fragments is as follows (2):

(2) $$\mathrm{dd-cfDNA}\left(\%\right)=\frac{C_{human\;male}\;}{C_{human\;small\;}-C_{human\;large}}\;*\;100\%$$

### 2.8. Clinical Utility

The clinical usefulness of the presented genetic approach will be evaluated both for each component step and for the general algorithm. Thus, the effectiveness of the use of the WES of donors and recipients will be assessed by the number (percentage) of cases of clinically significant genetic variants associated with end-stage heart diseases.

Moreover, a pharmacogenetic assessment will be carried out both for recipients and at the epidemiology level of the carriage of significant genetic variants in the presented sample of donors and recipients.

The diagnostic utility of determining the ratio of dd-cfDNA by dPCR will be evaluated by comparing the results obtained with clinical data from the recipients.

### 2.9. Data Analysis

All statistical analyses will be performed with the SPSS (version 20.0) and Jamovi (version 2.6.17) software. Descriptive statistics will be carried out with the calculation of the mean (M) and standard deviation (SD); percentages will be calculated for qualitative variables. Comparative analysis will be carried out using t (Student’s) criteria for independent and paired samples, ANOVA, and Bayesian analysis of variance. A statistically significant difference was accepted for a *p* value of less than 5%.

### 2.10. Ethical Considerations

This study was conducted in accordance with the Declaration of Helsinki and national ethical regulations governing biomedical research and organ transplantation in Kazakhstan. The study has been approved by the Local Bioethics Commission of the “University Medical Center” Corporate Fund (Protocol No. 3 dated 14 July 2023), and all necessary safeguards were implemented to ensure the protection of participants’ rights, confidentiality, and anonymity.

For heart and kidney transplant recipients, as well as living kidney donors, written informed consent was obtained either directly from the individuals or from their legal representatives. The written informed consent process was carried out at the University Medical Center CF and the National Research Oncology Center (Astana, Kazakhstan), where transplantation procedures and post-transplant follow-up were conducted.

In cases involving deceased heart donors, organ procurement and the use of biological materials were conducted in accordance with Kazakhstan’s legal framework. As per national guidelines and algorithms, donors’ biological samples were transferred to the Research and Production Center of Transfusiology for HLA typing to assess compatibility. Before the organ retrieval process, the legal representatives of the deceased donor provided written informed consent, which included authorization for the use of biological materials and test results in scientific and statistical research, with personal data protection ensured.

All study procedures adhered to international and national bioethical principles, ensuring the voluntary participation of individuals, respect for donor rights, and strict measures to maintain confidentiality and anonymity.

## 3. Preliminary Results

This study presents preliminary findings from donor–recipient pairs involving kidney and heart transplant cases, where the recipients were female, and the donors were male. Specifically, the dataset includes three HTx cases, five adult kidney transplant (AKTx) cases, and two pediatric kidney transplant (PKTx) cases. Among the study participants, no clinically confirmed cases of graft rejection were observed during the study period.

Overall, the WES data revealed read counts ranging from 41,077,830 to 49,492,614, with Q30 coverage of target regions ranging from 97.3% to 99.0%. The number of identified SNPs varied between 72,050 and 73,909.

### 3.1. Heart Transplantation

The average age of the HTx recipients at the time of transplantation ranged from 26 to 46 years. Among the cases, two patients were diagnosed with dilated cardiomyopathy (DCM), while one patient had hypertrophic cardiomyopathy (HCM). The mean time elapsed between transplantation and inclusion in the study was 69.3 ± 40.4 months (range: 25–104 months). Table 1 provides detailed clinical data, genotyping results, and donor-derived cell-free DNA (dd-cfDNA) percentages for HTx recipients.

In HTx recipients, a pathogenic mutation in the RBM20 gene was identified in one patient, while variants of uncertain significance (VUS) were detected in the TTN and PKP2 genes in two patients (Table 1).

The %dd-cfDNA levels, determined using Y-chromosome fragment analysis, varied among HTx recipients. In patients HTx1 and HTx3, the %dd-cfDNA levels ranged from 0.001 to 0.04%. However, patient HTx2 exhibited higher %dd-cfDNA levels, ranging from 0.46% to 0.80% (Table 1). Notably, there was a strong positive correlation between the time elapsed since transplantation and the mean %dd-cfDNA levels observed for all periods (r = 0.851, *p* < 0.001).

### 3.2. Adults Ans Pediatric Kidney Transplantation

Among the five AKTx recipients, four patients were diagnosed with glomerular diseases, and one patient had chronic kidney disease (CKD) secondary to hypertension. The average age of AKTx recipients was 50.0 ± 12.7 years. Of these, one patient received a cadaveric kidney, while the remaining four received kidneys from living related donors (Table 2).

Two cases of PKTx were included in the study, involving recipients aged 7 and 17 years. Both patients presented with congenital urinary tract abnormalities and received kidneys from living related donors.

Among both PKTx and AKTx recipients, no clinically significant genetic variants were identified. At the same time, the sequencing of donor genomes revealed a pathogenic mutation in the *UMOD* gene (c.326T > A; p.Val109Glu), associated with tubulointerstitial kidney disease, autosomal dominant, type 1 (OMIM #162000), in the donor of a PKTx recipient, and a VUS variant in the *APOL1* gene (c.29+1G > C), associated with focal segmental glomerulosclerosis 4 (OMIM #612551), in the donor of an AKTx recipient. These findings underscore the importance of monitoring both donors and recipients for potential long-term implications.

Among AKTx recipients from living donors, %dd-cfDNA levels on post-transplant day 3 ranged between 0.17% and 0.30%. In contrast, the recipient of a cadaveric kidney (AKTx2) exhibited significantly higher %dd-cfDNA levels of 12%. During subsequent follow-ups, %dd-cfDNA levels stabilized, ranging between 0.002% and 0.45%, regardless of the donor type (Table 2), which remained below previously established thresholds for diagnosing active rejection in KTx recipients (≥0.5–1%) [35,36].

In pediatric recipients, %dd-cfDNA levels showed marked variability. In patient PKTx1, the levels ranged between 0.07% and 0.23%. However, patient PKTx2 displayed substantially elevated %dd-cfDNA levels on days 3 and 14 post-transplantation, with readings of 1.72% and 7.35%, respectively. These elevated levels in PKTx2 were likely attributable to a severe post-transplantation infectious complication (Table 2). Additionally, genetic analysis revealed that PKTx2 was heterozygous for the variants rs1045642, rs2032582, rs1128503 (*ABCB1*), and rs3740066 (*ABCC2*), which are associated with tacrolimus metabolism and an increased risk of acute cellular rejection (Table 3) [35]. However, associations between pharmacogenetic analysis and data on metabolism, the adverse effects of immunosuppressive therapy, and post-transplant clinical outcomes should be further analyzed in future studies.

### 3.3. Pharmacogenetics

Variants in pharmacogenetically significant genes were identified, including *ABCB1*, *ABCC2*, *CYP2J2*, *CYP3A4*, *KCNJ11*, *NR1I2*, *POR*, and *SUMO4*. These variants are associated with tacrolimus metabolism, drug adverse effects, and the risk of graft rejection (Table 3). The identified genetic profiles highlight the necessity of the ongoing surveillance of transplant recipients and the need for further investigation into the role of these variants within the Kazakhstani population.

## 4. Discussion

This study presents a protocol integrating whole-exome sequencing (WES), pharmacogenetic profiling, and donor-derived cell-free DNA (dd-cfDNA) quantification as a personalized approach to transplantation medicine. Moreover, to demonstrate the feasibility of this approach, this study presents preliminary findings from both kidney and heart transplantation. The findings contribute to the growing body of knowledge on personalized approaches to transplantation medicine.

NGS technology allows its broad application to diverse areas in solid organ transplantation. Early studies have shown a high value of NGS in high-throughput and high-resolution human leukocyte antigen genotyping (histocompatibility), noninvasive monitoring of allograft rejection, and immune repertoire analysis [47,48]. The sequencing of HLA genes revealed variants and alleles associated with the pharmacogenetics of certain drugs (Carbamazepine, Vancomycin) and mortality after transplantation [47,49,50]. At the same time, the capabilities of the NGS are not limited to HLA typing. The NGS-based WES of recipients will allow for determining the possible genetic etiology of the end-stage organ disease, and, if necessary, to conduct genetic counseling to the recipient’s families in case of detection of genetic variants associated with the etiology of the disease. These results will provide data on the prevalence of genetic diseases leading to organ transplantation for the first time in Kazakhstan. The genomic profiling of organ donors may also contribute to a more comprehensive risk–benefit evaluation, providing evidence to refine donor selection criteria and long-term post-transplant monitoring strategies. However, the clinical utility of these approaches in transplantation medicine requires further validation in prospective studies.

To assess the feasibility of this protocol, we conducted a preliminary evaluation of donor–recipient pairs in heart (HTx) and kidney transplantation (KTx), focusing on genetic variants, pharmacogenetic markers, and dd-cfDNA levels. These findings are not intended as definitive conclusions but rather as an initial demonstration of the applicability of this approach.

The identification of pathogenic mutations and variants of uncertain significance (VUS) underscores the critical role of genomic analysis in transplantation. Among HTx recipients, a pathogenic mutation in the RBM20 gene was identified in one patient, while two others carried VUS in TTN and PKP2, which are genes associated with cardiomyopathies [51,52,53]. These findings suggest a possible genetic contribution to their pre-existing conditions and highlight the importance of thorough genetic screening among the recipient’s family members. In KTx recipients, the discovery of a UMOD mutation in the donor of a pediatric recipient and a VUS in APOL1 in an adult donor highlights the potential long-term risks for both donors and recipients. These variants are associated with tubulointerstitial kidney disease and focal segmental glomerulosclerosis, respectively, emphasizing the necessity of close monitoring. While these findings do not establish causality, they underscore the potential of integrating genetic data into pre- and post-transplant assessments.

Due to the fact that there is a large amount of data on potential predictors of metabolism and susceptibility to immunosuppressive drugs, WES will allow us to compare various genetic variants with the metabolic rate of the corresponding drug. Moreover, information will be obtained on the prevalence of certain genetic variants significant for pharmacogenetics in the Kazakhstani population. Thus, the detection of pharmacogenetically significant variants in genes such as *ABCB1*, *ABCC2*, *CYP2J2*, *CYP3A4*, *KCNJ11*, *NR1I2*, *POR*, and *SUMO4* further underscores the importance of tailoring immunosuppressive therapies. For instance, patient PKTx2’s heterozygosity for *ABCB1* and *ABCC2* variants, associated with tacrolimus metabolism and acute cellular rejection, likely contributed to the patient’s post-transplantation infection complications [37]. These preliminary findings suggest that pharmacogenetic testing may help tailor immunosuppressive regimens in future clinical applications. However, further validation is necessary to confirm the clinical impact of these associations.

There are various dd-cfDNA detection methods to assess the risk of transplant rejection. In terms of methodology, two broad strategies are employed, involving the differentiation of donor and recipient cell-free DNA (cfDNA) through either random or targeted means. This differentiation is achieved through diverse techniques, such as NGS, Droplet Digital PCR (ddPCR) or quantitative real-time PCR (qPCR). Several commercial solutions, namely TRAC, TheraSure, AlloSure, and Prospera, are accessible for this purpose [54,55,56,57,58]. In this study, we planned to evaluate the feasibility and diagnostic potential of allele-specific ddPCR for dd-cfDNA quantification in solid organ transplantation.

In our preliminary assessment, dd-cfDNA levels were generally low among HTx recipients, except in one patient (HTx2), who showed transiently elevated levels (0.46–0.80%), despite no signs of rejection. Previous studies demonstrated that a threshold of 0.25%/0.2%/0.1% results in a 99%/97.1%/99% negative predictive value (NPV), respectively [59,60,61]. This highlights the importance of contextualizing dd-cfDNA findings with clinical and histological data to avoid over-interpretation.

In KTx recipients, cadaveric donors were associated with markedly higher %dd-cfDNA levels in early post-transplantation (e.g., 12% in AKTx2) compared to living donors. This observation aligns with prior study reporting elevated %dd-cfDNA levels in deceased-donor compared to living-donor kidney recipients [62]. The acute spike in %dd-cfDNA levels, as seen in AKTx2 (12%), underscores the impact of donor type on early post-transplant molecular dynamics. However, this early elevation gradually declined over time, reinforcing the need for longitudinal monitoring to determine whether %dd-cfDNA trajectories can reliably distinguish between normal recovery and rejection risk.

Previous studies applied thresholds of dd-cfDNA levels ≥1%, ≥0.74% and ≥0.5% for diagnosing active rejection in KTx recipients [35,36]. In our case, the elevated %dd-cfDNA levels observed in pediatric patient PKTx2 (1.72% and 7.35% on days 3 and 14, respectively) were likely attributable to severe infectious complications. This aligns with prior research showing elevated dd-cfDNA levels in transplant recipients with infections, including CMV and BK virus nephropathy [63,64]. These findings further support the need for the cautious interpretation of dd-cfDNA results in clinical practice, particularly in the early post-transplant period, when multiple confounding factors may influence biomarker levels.

Thus, the authors suggest that the presented algorithm of genetic diagnosis, including whole-exome sequencing followed by dd-cfDNA determination with the dPCR method, will expand the possibilities of the genetic counseling of donors and recipients, as well as increase the effectiveness of the use of molecular genetics in the study of the etiology of the disease, pharmacogenetics, and patient monitoring using an integrated personalized approach in transplantation medicine.

Although these preliminary findings provide an initial demonstration of the study protocol’s feasibility, they are subject to several limitations. The small sample size, lack of longitudinal follow-up, and absence of biopsy-confirmed rejection cases prevent definitive conclusions on the clinical applicability of genetic and molecular biomarker-based strategies. Additionally, dd-cfDNA levels can be influenced by multiple confounding factors (e.g., infections, medication adherence, comorbidities), which were not fully accounted for in this preliminary dataset. Despite these limitations, this study presents a novel, protocol-driven approach to integrating genomic and molecular profiling into solid organ transplantation, laying the foundation for future research aimed at improving long-term graft survival and patient outcomes. Future studies should aim to expand the sample size to validate these initial findings, while incorporating prospective, longitudinal monitoring to assess the predictive value of genetic markers, pharmacogenetic variants, and dd-cfDNA trends in transplant outcomes. Additionally, research should focus on developing integrative models that combine molecular biomarkers with clinical parameters, enabling a more comprehensive and personalized approach to post-transplant management.

## Figures and Tables

**Table 1:** Heart recipients: clinical data, genotyping, and dd-cfDNA%.

Case	Diagnosis	Age at HTx (Year)	Time (Month)	Genetic Variant	ACMG	dd-cfDNA %					
						T1	T2	T3	T4	M ± SD	F, p
HTx1	DCM	33	79	*TTN*:c.29338A > G (p.Ile9780Val)	VUS	0.04%	0.04%	0.01%	0.0001%	0.023 ± 0.021	34.0, *p* = 0.003 Post-hoc test: HTx2 vs. HTx1/HTx3, *p* < 0.05
HTx2	DCM	26	104	*RBM20*:c.1907G > A (p.Arg636His)	P	0.70%	0.60%	0.80%	0.46%	0.640 ± 0.145	
HTx3	HCM	46	25	*PKP2*:c.288T > G (p.Asp96Glu)	VUS	0.008%	0.006%	0.002%		0.005 ± 0.003	

HTx—heart transplantation; Time—time period between HTx and first assessment of dd-cfDNA in months; DCM—dilated cardiomyopathy; HCM—hypertrophic cardiomyopathy; P—pathogenic; VUS—variant of uncertain significance.

**Table 2:** Adult and pediatric kidney recipients: clinical data, genotyping, and dd-cfDNA%.

Case	Diagnosis	Age at KTx (Year)	Donor Type	dd-cfDNA %				
				T1	T2	T3	T4	T5
AKTx1	Glomerular disease	66	LDKT	0.17%	0.08%	0.13%	0.03%	0.002%
AKTx2	CKD	51	DDKT	12.0%	0.60%	0.20%	-	0.10%
AKTx3	Glomerular disease	57	LDKT	0.24%	0.17%	0.06%	0.09%	0.25%
AKTx4	Glomerular disease	43	LDKT	0.17%	0.45%	0.12%	-	-
AKTx5	Glomerular disease	33	LDKT	0.30%	0.02%	-	-	-
PKTx1	CAKUT	17	LDKT	0.07%	0.17%	0.19%	0.23%	0.23%
PKTx2	CAKUT	7	LDKT	1.72%	7.35%	-	0.51%	-

AKTx—adult kidney transplantation; PKTx—pediatric kidney transplantation; CKD—chronic kidney disease; CAKUT—congenital anomalies of the kidney and urinary tract; LDKT—living-donor kidney transplantation; DDKT—deceased-donor kidney transplantation.

**Table 3:** Identified genetic variants with pharmacogenetic significance for tacrolimus prescribing.

Genetic Variant (rs)	Gene	Distribution Among 10 Study Participants	Effect	Reference
rs1045642	*ABCB1*	6 heterozygous and 2 homozygous cases	SNPs were found to have a potential effect on early tacrolimus C0/D	[37]
rs2032582	*ABCB1*	5 heterozygous and 3 homozygous cases		
rs1128503	*ABCB1*	4 heterozygous and 3 homozygous cases	Association with acute cellular rejection	[38]
rs2229109	*ABCB1*	1 heterozygous case	Association between SNP and tacrolimus intracellular accumulation	[39]
rs3740066	*ABCC2*	3 heterozygous cases	SNP was found to have potential effect on early tacrolimus C 0/D	[37]
rs717620	*ABCC2*	1 heterozygous case	Significant factor of tacrolimus lnC/D among LTx recipients	[40]
rs890293	*CYP2J2*	1 heterozygous case	Influenced the renal function of these patients and the occurrence of adverse events during treatment with tacrolimus among KTx recipients	[41]
rs2242480	*CYP3A4*	1 heterozygous case	Carriers had an almost twofold increase in the tacrolimus C0/D compared to that of the non-carriers	[42]
rs5219	*KCNJ11*	4 heterozygous cases	Polymorphism associated with a new-onset posttransplant diabetes in patients treated with tacrolimus	[43]
rs2276707	*NR1I2*	5 heterozygous cases	Impact tacrolimus clearance in kidney and liver transplant recipients	[44]
rs1057868	*POR*	4 heterozygous and 1 homozygous cases	SNPs rs1057868-rs2868177 GC-GT diplotype is associated with high tacrolimus concentrations in early post-renal transplant recipients	[45]
rs237025	*SUMO4*	8 heterozygous and 1 homozygous cases	SNP contributes to the development of new-onset diabetes mellitus after liver transplantation	[46]

## Data Availability

The raw data supporting the conclusions of this article will be made available by the authors on request.
